# Safety and Efficacy of Extremely Low LDL-Cholesterol Levels and Its Prospects in Hyperlipidemia Management

**DOI:** 10.1155/2018/8598054

**Published:** 2018-04-23

**Authors:** Dhrubajyoti Bandyopadhyay, Arshna Qureshi, Sudeshna Ghosh, Kumar Ashish, Lyndsey R. Heise, Adrija Hajra, Raktim K. Ghosh

**Affiliations:** ^1^Department of Internal Medicine, Mount Sinai St Luke's Roosevelt, New York, NY, USA; ^2^Department of Medicine, Lady Hardinge Medical College, New Delhi, India; ^3^IPGMER, Kolkata, India; ^4^The University of Texas MD Anderson Cancer Center, Houston, TX, USA; ^5^Department of Internal Medicine, University of Nebraska Medical Center, Omaha, NE, USA; ^6^Department of Internal Medicine, IPGMER, Kolkata, India; ^7^Division of Cardiovascular Diseases, Metrohealth Medical Center, Case Western Reserve University, Cleveland, OH, USA

## Abstract

The risk of cardiovascular disease has been reported to have a linear relationship with LDL levels. Additionally, the currently recommended LDL target goal of 70 mg/dl does not diminish the CV risk entirely leaving behind some residual risk. Previous attempts to maximally lower the LDL levels with statin monotherapy have met dejection due to the increased side effects associated with the treatment. Nevertheless, with the new advancements in clinical medicine, it has now become possible to bring down the LDL levels to as low as 15 mg/dl using PCSK9 monoclonal antibodies alone or in combination with statins. The development of inclisiran, siRNA silencer targeting PCSK9 gene, is a one step forward in these endeavors. Moreover, various studies aiming to lower the CV risk and mortality by lowering LDL levels have demonstrated encouraging results. The current challenge is to explore this arena to redefine the target LDL levels, if required, to avoid any suboptimal treatment. After thorough literature search in the PubMed, Embase, Scopus, and Google Scholar, we present this article to provide a brief overview of the safety and efficacy of lowering LDL below the current goal.

## 1. Introduction

Hyperlipidemia has always been a topic of interest owing to the concomitant increased risk of adverse cardiovascular events. Coronary artery disease, a leading cause of death in the United States with almost 400,000 deaths/year, is found to be strongly associated with hyperlipidemia [1]. Moreover, increased LDL levels are found to be positively correlated with the increased CV risk. Thus, the treatment of hyperlipidemia plays a crucial role in the management of patients with CAD or those at increased risk of CAD all around the world. About 73.5 million adults in the USA have elevated LDL-cholesterol [2]. The American College of Cardiology/American Heart Association (NCEP IV) guidelines recommend prescription of evidence-based doses of statins independent of the LDL level [3].

Interestingly, most physicians prefer treating to an LDL goal and consider 70 mg/dl to be an appropriate target goal for people at the highest risk for cardiovascular disease [4]. However, despite achieving the target level of 70 mg/dl with high-intensity statin therapy, there is residual CV risk. Furthermore, targeting HDL and TG levels to reduce this residual risk has been proved futile [5]. Meanwhile, the recent availability of PCSK9 inhibitors has revalidated the discussion on further lowering of LDL and has brought back the age-old question: how low is in fact low enough to bring the CV risk to the minimum?

## 2. LDL Metabolism and Pathophysiology of Atherosclerosis

**Figure 1:**
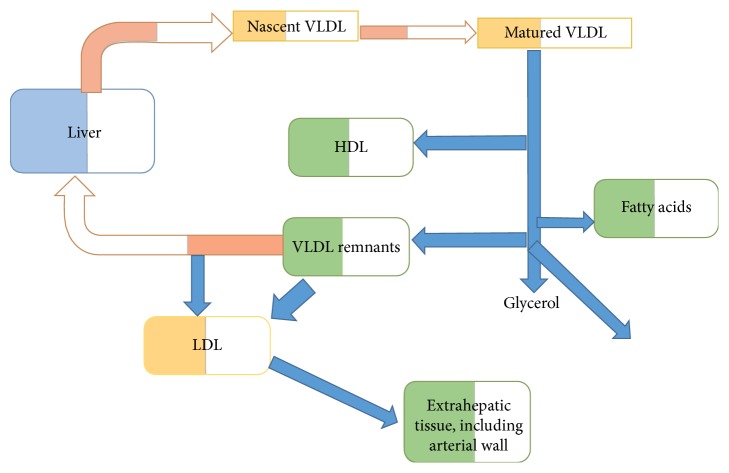
LDL metabolism.

The level of LDL is the single most important marker of atherosclerosis (Figure 1). Deranged LDL metabolism leads to coronary artery disease that is often fatal, especially in patients with diabetes. It has been found that not only elevated levels of LDL lead to coronary heart disease, but changes in composition can also result in the same. As we all know, cholesterol is an integral part of the plasma membrane, and a minimum level of LDL needs to be present to maintain structural integrity and sustain normal function of cells.

**Figure 2:**
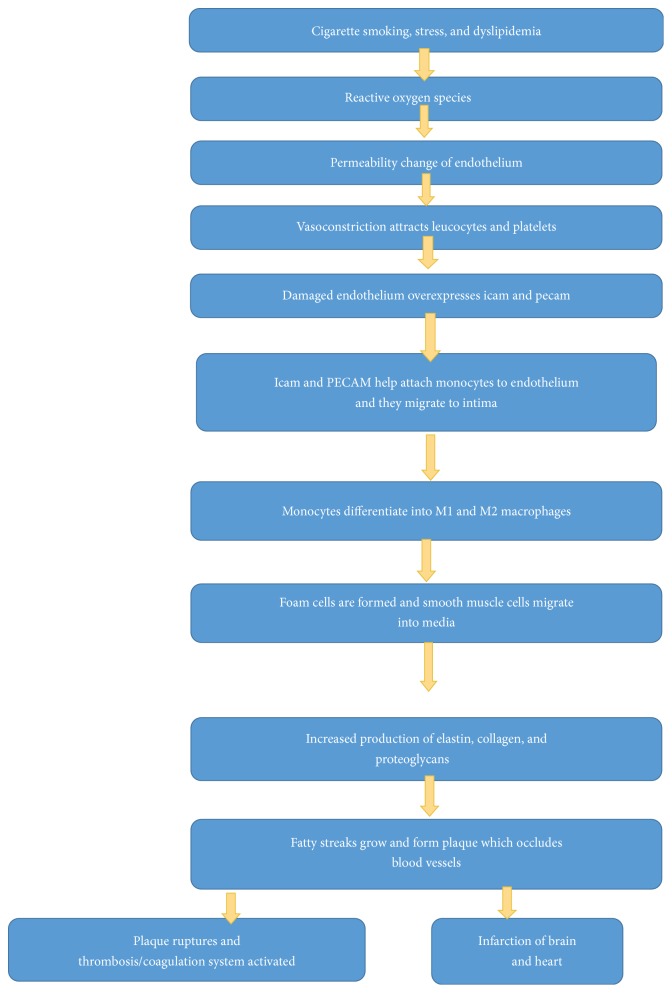
Mechanism of atherosclerosis.

The development of atherosclerosis is indeed a complicated process where LDL plays a pivotal role. LDL causes endothelial damage which helps in the progression and formation of fatty streaks. Atherosclerosis, the most important factor behind the coronary vascular disease, affecting mostly medium- and large-sized arteries is characterized by the presence of modified smooth muscles, foam cells, endothelial cells, WBCs, and lipid in the center. With the growing comprehension of inflammatory process and mediators, studies have revealed that lipid-related inflammation could be cornerstone mediator for atherosclerosis [6] (Figure 2). The most likely site for plaque formation is the regions that experience low endothelial stress rather than area experiencing high stress. The plaques continue to grow into the lumen, and they experience increasingly high stress as the lumen diameter becomes narrower which ultimately contributes to the destabilization of the plaque [7]. Atherosclerosis can be prevented by implementing lifestyle modifications, controlling the risk factors of which controlling high LDL is of paramount importance.

## 3. Commonly Used LDL-Lowering Drugs

*Statins. *Statins inhibit the HMG-CoA reductase enzyme, the rate-controlling enzyme in the biosynthesis of cholesterol [8]. Statins lower LDL-C and triglycerides while slightly raising HDL. It is the standard of care for dyslipidemia management. Liver damage, muscle pain, and increased risk of type 2 diabetes mellitus are some of the side effects of statins, but benefits outweigh the risks [9]. They also prevent SMC migration and proliferation and impede the activation of TNF-alpha, IL-1 beta, and other interleukins which play an active role in inflammation [8].

*Ezetimibe. *This medication prevents the absorption of bile acid in the small intestine, lowers LDL, increases HDL slightly, and, to a little extent, lowers triglycerides. However, ezetimibe can cause myalgia and abdominal pain [10].

*PCSK-9 Inhibitors. *Proprotein Convertase Subtilisin/Kexin Type 9 (PCSK9) causes degradation of LDL receptors in the liver. Alirocumab and evolocumab are the two monoclonal antibodies directed against PCSK-9, and thus it prevents degradation of LDL receptors in the liver. Nasopharyngitis, reactions at the injection sites, flu-like symptoms, and muscle soreness are a few of the side effects that have been reported in the patients treated with alirocumab [11].

Fibrates, bile acid binding resins, and niacin are also used for lowering LDL-cholesterol.

## 4. The Historical Perspective of Extremely Low LDL Level

Individuals with hypobetalipoproteinemia and PCSK9 mutation have inherited natural protection from CAD. It is because of low LDL and consequently lower incidence of atherosclerosis and associated events. Patients with a total deficiency of PCSK9 have been reported to have LDL-C levels in the range of 15 mg/dl without having any adverse effects from these extremely low LDL levels [12].

Anthropological and historical evidence showed that, nearly 10,000 years ago, our ancestor hunter-gatherers, who were primarily dependent on wildlife diet which was mostly nuts, fruits, vegetables, and flesh of wild animals, were free from atherosclerosis with an average cholesterol level 50–75 mg/dl. Only after 500 generations, after the agricultural revolution, the modern day evolved human beings are mostly reliant on processed food, refined sugars, and carbohydrates. Even the meat that we consume today is obtained from animals which are fed processed grains and corns that make the meat deficient in omega-3 fatty acids. In this short period, the massive changes in our dietary habits took place, which is not long enough for the genetic adaptations to happen to handle this excess load of cholesterol and this causes the rise of average serum cholesterol level to somewhat around 220–230 mg/dl. These findings suggest drastic changes in our diet in comparison to genetic adaptations which are somewhat responsible for the rise of serum LDL level and increased incidence of atherosclerotic diseases [13]. Also, we know about South Asian paradox which denotes that South Asian people are more prone to develop CAD despite having within-target LDL level. So, we have to consider whether further lowering of LDL below the existing target level would help in reduction of atherosclerosis burden and CV events in those cases [14].

## 5. What Defines Low and Extremely Low LDL and Its Proposed Benefits and Adverse Effects

The LDL-C level of less than 50 mg/dl is considered low while a level of less than 20 mg/dl is considered extremely low. Intensive lipid lowering treatment has been found to halt the progression of atherosclerosis as compared to moderate lipid lowering treatment. It also regresses the atheroma plaque volume as reported by the REVERSAL (Reversal of Atherosclerosis with Aggressive Lipid Lowering) trial and ASTEROID (A Study to Evaluate the Effect of Rosuvastatin on Intravascular Ultrasound-Derived Coronary Atheroma Burden) trial. SATURN (Study of Coronary Atheroma by Intravascular Ultrasound: Effect of Rosuvastatin Versus Atorvastatin) trial also supports this [15]. An LDL level below 2.5 mmol/l can cause an atherosclerotic plaque to regress. Similarly, GLAGOV trial reported that patients who received evolocumab on a baseline treatment with statins demonstrated plaque regression in a larger number of patients as compared to placebo (64.3% versus 47.3%) after 76 weeks of therapy [16]. In a retrospective analysis, coronary calcium score was reduced with the aggressive lowering of LDL [17].

Though the intensive lowering of LDL reduces the plaque size, there is an ongoing debate regarding its side effects. Few previous clinical trials had reported increased incidences of adverse events such as hemorrhagic strokes, dementia, depression, hematuria, and cancers with extremely low LDL-C. The Dallas Heart Study (*n* = 12887), a population-based study, stretched over a period of 15 years found that a PCSK9 mutation is associated with significantly low LDL level. People with PCSK9 mutation exhibited a low incidence of CAD (a reduction of 88 percent in black and 47 percent of whites) with no increase in the hemorrhagic stroke or cancer. A person with a complete absence of PCSK9 has LDL level of about 15 mg/dl, and there has not been any report of any adverse incidents [18]. The brain itself contains 25% of total cholesterol, and it is needed for maintaining its complex neuronal circuit. Blood-brain barrier is impermeable to circulatory cholesterol. This fact implies that the cholesterol regulation in the brain is not similar to that of extracerebral cholesterol. So, cholesterol level outside of the brain should not affect the brain functioning as these two cholesterol pools are different. On the surface, a target LDL level of less than 70 mg/dl may appear markedly low, but its cogency can be supported by a physiological rationale that we are born with an LDL level of 30–40 mg/dl, and, at that time, the development of the brain is at its peak. The safety of low LDL level has been supported by Ray et al. They reported that the reduction of LDL levels to as low as that of a neonate is safe as well as beneficial in reducing the risk of angina, MI, or cerebrovascular disorder and total mortality [19]. These might be the levels to which humans are inherently adapted, and the levels ventured to be achieved [14]. Human brain is the most cholesterol-enriched organ but, unlike other peripheral organs, human brain is primarily dependent on de novo cholesterol synthesis rather than peripheral plasma cholesterol [20]. These above pieces of evidence lead to the hypothesis that the lowering of plasma LDL would not affect the normal brain function.

## 6. Why Extremely Low or Low LDL Is Now Discussed

Most of the statin trials showed an average of 31% of relative risk reduction which means that 69% of relative risk is still present. Despite the widespread use of statins, cardiovascular diseases and strokes are responsible for 25% of deaths worldwide. There is certainly need to address this residual risk. A meta-analysis by the Cholesterol Treatment Trialists (CTT) contributors reported that a reduction of 1 mmol per liter in LDL-cholesterol levels results in a consistent 20% to 25% decrease in the risk of the major cardiovascular events as well as the total mortality decreasing by 12 percent [21]. PROVE IT-TIMI study noted a residual CV risk of 22.4% despite reducing LDL-C to 62 mg/dl. This residual risk was targeted in various studies by modulating HDL and TG levels but showed disappointing results. However, recently PCSK9 inhibitors are emerging as a promising alternative to achieve LDL levels even below the target. Statin monotherapy upregulates the PCSK9 by 25–35% on average, along with LDL receptors in hepatocytes, which counterbalances the beneficial effects of statin. Thus, PCSK9 inhibitors would also mitigate the intrinsic counterbalancing effect of statins when given in combination [22]. However, the dilemma that continues to trouble physicians is determining how aggressively LDL needs to be treated. After the recent pooled analysis of 14 trials by Robinson et al. which showed the safety and efficacy of alirocumab in attaining low LDL level even below 15 mg/dl, this topic gains momentum [23].

## 7. Studies Showed Promising Results of Extremely Low LDL/Low LDL Level and Safety

Many trials piloted to ascertain the effects of lower-than-recommended LDL levels have reported promising results (Table 1). The TNT Trial was conducted to investigate the impact of very low LDL-C levels on major cardiovascular events compared with relatively higher LDL-C levels. The study revealed a highly significant reduction in the rate of major cardiovascular events with descending levels of LDL-cholesterol (*p* < 0.0001) with a decrease of 22 percent in combined cardiovascular end point (including coronary artery disease, nonfatal MI, and resuscitated cardiac arrest and a reduction of 20 percent in cardiac deaths with lower LDL levels) [24]. Additionally, the dreaded side effects of a very low LDL-C level such as muscle pain, hemorrhagic stroke, and death due to cancer were not increased. In the IMPROVE-IT (The Improved Reduction of Outcomes: Vytorin Efficacy International Trial), 18,144 participants with acute coronary syndrome were assigned to either simvastatin (40 mg) plus ezetimibe (10 mg) or simvastatin (40 mg) plus placebo randomly. At seven years, the rate of combined cardiovascular death, major coronary event (nonfatal myocardial infarction, unstable angina, or coronary revascularization), or nonfatal stroke was significantly lower in the simvastatin-plus-ezetimibe group (32.7% versus 34.7%). It was observed in those who had baseline LDL level well below the current LDL goal [25]. In a study in 2007, a statin was prescribed to a group of patients with LDL-C less than 60 mg/dl who also had other comorbid conditions such as diabetes mellitus or ischemic heart disease. After a follow-up period of 2.0 +/− 1.4 years, it was found that statin improved survival not only in patients taking them at the baseline level (HR, 0.58; 95% CI, 0.38 to 0.88) but also in those who have LDL-C below 40 mg/dl. Even patients without ischemic heart disease showed improved survival. However, there was no increased risk of elevated transaminases, malignancy, or rhabdomyolysis [26].

The JUPITER trial compared the clinical outcomes and adverse events in patients treated with rosuvastatin who attained LDL-C less than 50 mg/dl and those who did not. The study revealed reduced major cardiovascular events by 65% among those attaining LDL-C < 50 mg/dl and by 44% for the rest of the cohort. Similarly, all-cause mortality was decreased by 46% among patients achieving LDL-C < 50 mg/dl and by 20% for the remaining cohort. However, there was also a higher rate of adverse events including diabetes, hepatobiliary disorders, and insomnia in patients with LDL-C < 30 mg/dl [27]. The SPARCL (Stroke Prevention by Aggressive Reduction in Cholesterol Levels) study conducted on patients with stroke or transient ischemic attack with atorvastatin 80 mg found that the statin reduced the chances of stroke in these groups of patients but increased the incidence of hemorrhagic stroke [28]. On the contrary, another study carried out among subjects with a history of myocardial infarction who were treated with either 80 mg simvastatin as a part of intensive statin therapy or 20 mg simvastatin reported no difference regarding hemorrhagic stroke after a mean follow-up period of 6.7 years (SD of 1.5 years). However, myopathy cases were reported in a higher number, among 80 mg simvastatin users [29]. Robinson et al. evaluated the safety of alirocumab. They described LDL-C levels to be as low as 15 mg/dl and did not report any adverse neurocognitive event, although a nonsignificant increase in cataract incidence seemed to be more in the group achieving LDL-C levels < 25 mg/dl [23]. In the same vein, Sabatine et al. did not report any significant increase in adverse reactions with very low LDL-C. Also, Prostate Cancer Prevention Trial indicated that low cholesterol is associated with reduced risk of high grade of prostate cancer [30]. A Retrospective Observational Study conducted in Quebec, Canada, on patients admitted with acute myocardial infarction concluded that high dose statin use might be associated with significant reduction in the cancer incidence [31]. The substudy of PROVE IT-TIMI 22 investigating 80 mg atorvastatin versus 40 mg pravastatin also proved that achieving LDL-C level below the expected level (80 to 100 mg/dl) is not associated with increased adverse events [32].

A meta-analysis by Boekholdt et al. reported an increased risk of hemorrhagic stroke in those with very low levels of LDL as compared to moderately low levels. However, the absolute number was low, and the statistical power was therefore insufficient to draw a definite conclusion. Furthermore, they believed that significantly lower risk of cerebrovascular events outweighed the potential for hemorrhagic stroke [33]. A Phase 3 Study of Evolocumab showed no increase in the adverse events despite a median LDL level of 26–36 mg/dl over 12 weeks [34]. The most recent data about safety and efficacy of low LDL comes from the FOURIER trial. They have shown a significant reduction of LDL from a baseline value of 92 mg/dl to 30 mg/dl with evolocumab. Most importantly, there was a significant decrease in the risk of major cardiovascular events without any major rise in the adverse events. They reported a 17% decrease in the cardiovascular death, myocardial infarction, and stroke on lowering the LDL to 43 mg/dl while reducing the LDL levels further to 22 mg/dl decreased the risk to 20%. Additionally, they reported consistent clinical improvements per unit reduction in LDL [35]. A post hoc analysis of 10 ODYSSEY trials comparing alirocumab with the control indicated that low LDL-C was associated with a lower incidence of major adverse cardiovascular events with no significant increment in the treatment-emergent adverse reactions [19].

Very recently, a prespecified safety analysis of IMPROVE-IT involving 15* *281 patients showed that patients (*n* = 971) with LDL level below 30 mg/dl had no increased adverse events over six years' follow-up [36].

## 8. Future Directions and Ongoing Studies

Low and extremely low LDL-C levels are being supported widely; however, many have raised concerns about their long-term effects which still stands unexplored. The mystery behind the advantages and disadvantages of prolonged exposure to pharmacologically induced low LDL levels needs to be unveiled.

A common finding among the LDL-lowering trials was the time lag between the onset of LDL lowering and the appearance of full clinical benefits regarding risk reduction presenting itself as another lacuna in the better understanding of the link between LDL-C lowering and CV risk reduction.

Bringing LDL to very low levels with statin monotherapy poses safety concerns in some patients. While the emergence of PCSK9 inhibitors has appeared to solve the problem, the cost effectiveness of treatment with PCSK9 inhibitors remains questionable. The immunogenic effects of the PCSK9 inhibitors varying from mild injection site reactions to anaphylaxis and loss of drug efficacy need to be scrutinized further. Recently SPIRE trial showed antibodies against the murine component of bococizumab in 15–20% of patients and a neutralizing antidrug antibody was seen only in 1.3% patients on alirocumab [37]. Poor adherence to the treatment due to multiple injections is another issue with the PCSK9 monoclonal antibodies.

A therapeutic strategy involving small (21–25 bp) interfering RNA (siRNA) targeting PCSK9 has gained our attraction recent past. Inclisiran, a novel therapeutic drug that inhibits PCSK9 through RNA interference, has shown encouraging results in an average reduction of LDL by 51% with only a 2-dose regime over a period of 9 months. This was investigated in a clinical phase 2 trial, ORION-1. The result of this trial is encouraging as ease of using this drug will be impactful as it needs only one or two injections over six-month to 1-year period [38]. Nevertheless, the impact on cardiovascular outcomes is yet to be studied in ORION-4. None of the studies have thus far mentioned the duration of treatment with PCSK9 inhibitors needed to maintain the risk reduction.

Peptide-based anti-PCSK9 vaccines are being tested on mice which supposedly have the potential to control LDL level for a longer duration [39].

Several ongoing studies are aiming to enhance our knowledge regarding the safety of low LDL and reduction in cardiovascular risk. In ODYSSEY OUTCOMES, a placebo-controlled phase 3 trial, 18600 post-MI patients are being randomized to alirocumab or placebo arm. It intends to compare the effects of alirocumab with placebo on the occurrence of cardiovascular events over a period of 64 months. The ODYSSEY APPRISE is a multicountry, multicenter phase 3 study aimed at investigating the safety of alirocumab in patients with severe hypercholesterolemia over a period of 30 months. TAUSSIG is another ongoing study designed to assess the long-term safety, tolerability, and efficacy of evolocumab in patients with severe hypercholesterolemia. Meanwhile, PACMAN-AMI is evaluating the effects of PCSK9 inhibition on the morphology of coronary plaque in patients with acute myocardial infarction.

## 9. Conclusion

In summary, the residual risk despite achieving the current target LDL levels needs to be addressed. Although the clinical benefits of lowering LDL have been well stated, their long-term consequences are still under investigation. Many trials conducted in the past were successful in reducing the LDL levels well below the target with a consequent reduction in CV risk. Though there is ample evidence that low LDL does protect from residual CV risk, there have also been a few studies claiming an increasing number of adverse events with low and extremely low LDL levels. Nevertheless, we have to wait for the result of ongoing trials to have a conclusive answer on the long-term effect of lowering the current LDL goal.

## Figures and Tables

**Table 1:** Summary of the trials that attained lower-than-recommended LDL level.

Trial, year of publication	*N*	Comparison	LDL reduction	CV event reduction	Adverse events	Comment
Cannon et al., 2004	4162	PVS versus AVS	-	26.3% versus 22.4%	21.4% versus 22.8%	Median LDL level - 62 mg/dl

TNT, 2005	18,003	AVS 10 mg versus 80 mg	-	20–30% fewer CV events in AVS 80 mg group	5.8% versus 8.1%	-

SPARCL, 2006	4731	AVS versus placebo	-	-	93% versus 91%	16% relative reduction in the risk of stroke

JUPITER, 2008	17,802	RSV versus placebo	50% lower LDL in RSV group	20% reduction for each 1 mmol/L decline in LDL level	15.2% versus 15.5%	Median LDL in RSV group of 55 g/dl at 12 months

IMPROVE-IT, 2015	18,144	EZE + statin versus statin	EZE lowered LDL-C further by 24%	7.2% lower risk of major vascular events	No significant difference	Mean LDL level I EZE group of 53.2 mg/dl

YUKAWA-2, 2016	404	E-mAb versus placebo	75.9% reduction in E-mAb group	-	46.5% versus 51%	Median LDL in E-mAb group of 28 g/dl

Post hoc analysis of 10 ODYSSEY trials, 2016	4974	A-mAb versus placebo, A-mAb versus EZE	−55.4% versus +2.7% −48.1% versus −18.0%	Every 39 mg/dl fall in LDL was translated into 24% lower risk of MACE	79.9% versus 81.3%, 76% versus 73.9%	33.1% of the pooled cohort achieved LDL < 50 mg/dl

Pooled analysis of 14 randomized trials, 2017	5234	A-mAb versus control (placebo or EZE)	LDL levels reported to be as low as 15 mg/dl in A-mAb group with an increase in adverse event rates	-	Low LDL levels (even <15 mg/dl) were not associated	-

FOURIER, 2017	27,564	E-mAb versus placebo	59% reduction as compared to placebo	-	No significant differences except injection site reactions were more common with E-mAb	Rate of CV events was 9.8% versus 11.3%

ORION-1 (dose ranging trial), 2017	501	Inclisiran versus placebo	51% reduction with 2 dose regimen	-	11% versus 8%	48% subjects attained LDL levels below 50 mg/dl

PVS: pravastatin, RSV: rosuvastatin, AVS: atorvastatin, EZE: ezetimibe, A-mAb: alirocumab, E-mAb: evolocumab, MACE: major adverse cardiac event.
